# Response adaptive randomisation in clinical trials: Current practice, gaps and future directions

**DOI:** 10.1177/09622802251348183

**Published:** 2025-06-18

**Authors:** Isabelle Wilson, Steven Julious, Christina Yap, Susan Todd, Munyaradzi Dimairo

**Affiliations:** 1 SCHARR, 7315 The University of Sheffield, UK; 2 Clinical Trials and Statistics Unit, 5053 Institute of Cancer Research, London, UK; 3 Department of Mathematics and Statistics, University of Reading, UK

**Keywords:** Response adaptive randomisation, outcome adaptive randomisation, adaptive design, adaptive allocation, randomised controlled trial, unequal treatment allocation, reporting

## Abstract

**Introduction:** Adaptive designs (ADs) offer clinical trials flexibility to modify design aspects based on accumulating interim data. Response adaptive randomisation (RAR) adjusts treatment allocation according to interim results, favouring promising treatments. Despite scientific appeal, RAR adoption lags behind other ADs. Understanding methods and applications could provide insights and resources and reveal future research needs. This study examines RAR application, trial results and achieved benefits, reporting gaps, statistical tools and concerns, while highlighting examples of effective practices. **Methods:** RAR trials with comparative efficacy, effectiveness or safety objectives, classified at least phase I/II, were identified via statistical literature, trial registries, statistical resources and researcher-knowledge. Search spanned until October 2023, including results until February 2024. Analysis was descriptive and narrative. **Results:** From 652 articles/trials screened, 65 planned RAR trials (11 platform trials) were identified, beginning in 1985 and gradually increasing through to 2023. Most trials were in oncology (25%) and drug-treatments (80%), with 63% led by US teams. Predominantly Phase II (62%) and multi-arm (63%), 85% used Bayesian methods, testing superiority hypotheses (86%). Binary outcomes appeared in 55%, with a median observation of 56 days. Bayesian RAR algorithms were applied in 83%. However, 71% of all trials lacked clear details on statistical implementation. Subgroup-level RAR was seen in 23% of trials. Allocation was restricted in 51%, and 88% was included a burn-in period. Most trials (85%) planned RAR alongside other adaptations. Of trials with results, 92% used RAR, but over 50% inadequately reported allocation changes. A mean 22% reduction in sample size was seen, with none over-allocating to ineffective arms. **Conclusion:** RAR has shown benefits in conditions like sepsis, COVID-19 and cancer, enhancing effective treatment allocation and saving resources. However, complexity, costs and simulation need limit wider adoption. This review highlights RAR's benefits and suggests enhancing statistical tools to encourage wider adoption in clinical research.

## 1. Introduction

In principle, regardless of the design used for a clinical study, trial integrity should be preserved, and the results from the trial should be valid and credible^1^ to influence practice positively. Adaptive designs (ADs) offer clinical trialists controlled flexibilities in the design and conduct of trials. They utilise accumulating outcome data to modify design aspects based on pre-specified decision-making criteria without undermining trial integrity and the validity of conclusions. Trial design aspects that can be modified include refining the original sample size, eliminating ineffective therapies, stopping the trial early when there is sufficient evidence to reach conclusions, and altering recruitment to target specific subgroups most likely to benefit from treatment.^2–4^

The use of ADs in practice has gradually increased over the years, although they are still not widely used in routine practice despite their prominence in the statistical literature.^5–7^ There is a disproportionate use of certain ADs, with some types, such as standard group sequential designs and, more recently, multi-arm multi-stage designs,^8^ being more prevalent than others.^9,10^ The potential to improve efficiency in evaluating treatment effects while balancing ethical and scientific interests makes ADs an attractive choice to researchers, funders, patients and the public.^11^

Response adaptive randomisation (RAR) is a type of AD that can be particularly useful when evaluating treatments in emergency care settings or for severe medical conditions. It helps balance the scientific interests of researchers and ethical interests of the patients and the public. Examples include the Ebola outbreak,^12^ the COVID-19 pandemic,^13^ and oncology settings.^14,15^ RAR provides the opportunity to update how incoming trial participants are allocated to treatments using methodology that gives more weight to favour the most promising and beneficial treatments as indicated by interim outcome data. As such, it is argued that RAR can target maximising the statistical power of a specific beneficial treatment and allocating more patients to treatments that show greater benefits, away from those that are less efficacious or potentially unsafe.^16^ In addition, it can be valuable in pruning futile or unsafe treatments from a basket of competing alternatives for further testing.^14^ It should be highlighted that the scope of RAR does not cover changes to allocation ratios solely as a consequence of triggering other trial adaptations that can occur when treatment arms are dropped, or new arms are added. For example, early stopping of arms (when unrelated to RAR) for efficacy or futility such as in multi-arm multi-stage trials technically updates the allocation ratios of dropped arms to 0 at interim analyses that trigger adaptation decisions.

**Figure 1.**
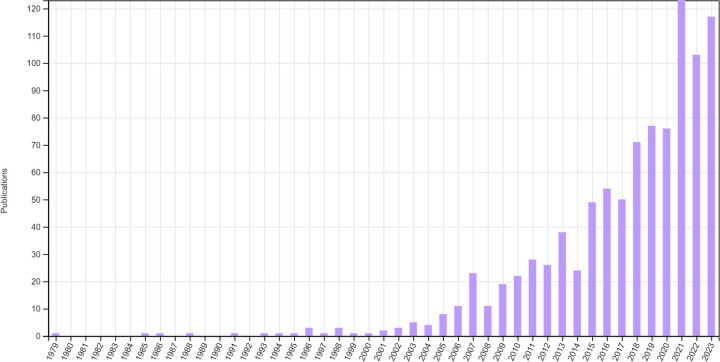
Publications relating to RAR identified from Web of Science up to the end of 2023.

Despite its appealing nature, the uptake of RAR in practice is thought to be disproportionately low, especially in confirmatory settings,^9,17^ particularly when compared to the theoretical interest it has generated since it was first proposed^18^ and the increase in the use of other ADs. Figure 1 displays the number of publications identified from Web of Science related to RAR (note that this is likely to be an incomplete picture as limited search terms were used, but the trend is evident). Possible explanations for this slow uptake could be attributed to a lack of practical knowledge,^19^ despite improvements in applied education over recent years,^10^ as well as some ongoing controversies^20^ and myths surrounding RAR.^16^

An in-depth understanding of how RAR has been applied in real trials could provide valuable educational insights and gaps that may need to be addressed to improve its appropriate uptake in the future. This study aims to identify clinical trials that were planned to use RAR methods and characterise their use with specific objectives to: (1) characterise the research context in which RAR is being applied; (2) provide an overview of RAR algorithms utilised, outlining specifics of trial design aspects; (3) explore what other trial adaptations are being implemented alongside RAR; (4) examine trial results presented, trial changes that were made, whether RAR was implemented as planned, and whether there were quantifiable benefits achieved by implementing RAR; (5) identify any gaps in the reporting of RAR aspects relating to design and conduct that may impede the comprehension of RAR methods and the interpretation of results by research consumers; (6) identify any concerns discussed about the use of RAR; (7) identify the statistical resources employed in implementing these RAR designs, such as code, packages or software; (8) highlight exemplars of good practice in the application of RAR.

## 2. Methods

This section briefly outlines the methods used to identify, select and analyse data from relevant clinical trials. Detailed methods are accessible via an open-access and prespecified protocol.^21^ No ethics approval was required for this research.

### 2.1 Eligibility screening

Completed or ongoing randomised trials investigating treatments in humans at the time of the search were eligible if they planned to use RAR at the design stage. That is, changes to allocation ratios for the next participant or cohort of participants must have been planned to be influenced by interim outcome data observed so far. This excluded trials that applied changes to allocation ratios solely based on interim baseline characteristics (e.g., using minimisation randomisation). Trials were also excluded where changes to allocation ratios were a consequence of other trial adaptations outside the scope of RAR (e.g., dropping or adding new arms), which can happen in multi-arm multi-stage or platform trials.

In addition, trials were included if they met the following inclusion criteria. They had comparative objectives (efficacy, effectiveness or safety) with at least two treatment arms including a comparator, were classified as phase I/II or higher or unclassified (common for non-pharmacological treatments such as devices or behavioural therapies), were within the access period up to the search date, had accessible full text (e.g., protocol, statistical analysis plan (SAP), results) or classification as RAR via other records (e.g., a clinical trial registry or abstract), and were written in English. Trials were excluded if they were classed as phase I or non-comparative. The decision to exclude phase I trials was because the majority of early phase trials are not randomised,^22^ and recent reviews did not identify any early phase RAR trials.^4,22^ Finally, trials without accessible full text and no clear classification as RAR on accessible records (e.g., a clinical trial registry or abstract) were also excluded.

### 2.2 Information sources

Data were obtained from four sources. First, relevant published trials were retrieved from previously completed searches of methodological statistical literature conducted as part of this research project (search strategy 1); more information can be found in the protocol.^21^ These searches were conducted on MEDLINE via PubMed, Embase via OVID and Web of Science. The searches on these three databases were last conducted in March 2023.

Second, supplementary searches were performed on two major clinical trial registries (search strategy 2): ClinicalTrials.gov (https://clinicaltrials.gov/) and the World Health Organisation (WHO) International Clinical Trial Registry Platform (ICTRP) (https://trialsearch.who.int). The searches for WHO ICTRP and ClinicalTrials.gov were last conducted on the 17th and 20th of October 2023, respectively.

Third, relevant published trials were retrieved from searches of statistical implementation resources (search strategy 3). GitHub (https://github.com/) and METACRAN (https://www.r-pkg.org/) were searched on the 5th and 9th of April 2024, respectively. As search strategy 3 took place after search strategy 1 and 2, it had the potential to pick up trials outside of the search window. Therefore, we decided to truncate this search to match search strategy 2's window (up to October 2023).

**Figure 2.**
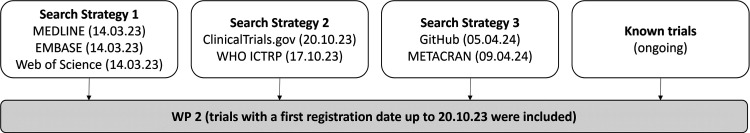
Information sources flow diagram.

Fourth, any other known eligible trials, including those known to researchers (e.g., from related work), were also included. Any trials identified outside this window (post-October 2023) are reported in Supplementary Materials, Table 10. See Figure 2 for a summary of the different information sources.

This approach allowed the identification of trials, including accessible protocols and SAPs that were not previously identified through MEDLINE via PubMed, Embase via OVID and Web of Science. Although trials must have been identified within the search period (October 2023), trial results that were known after this period were included (until 29 February 2024).

### 2.3 Search strategy

The protocol^21^ details the search strategy used for the statistical methodology; the same search terms were used to identify clinical trials. In the context of clinical trials, the two designated databases were searched for completed and ongoing trials. ClinicalTrials.gov and WHO ICTRP underwent similar searches, using the ‘other terms’ search bar and the search portal, respectively.

A scoping review of literature on MEDLINE and of grey literature on Google Scholar helped inform the search strategy. A list of all known terms used to refer to RAR was also compiled. An example of the search terms devised is provided: “adaptive randomisation” OR “adaptive randomization” OR “adaptive allocation” OR “adaptive treatment allocation” OR “adaptive treatment randomisation” OR “adaptive treatment randomization” OR “response adaptive” OR “responsive adaptive” OR “outcome adaptive” OR “adaptive design” OR “adaptive trial”.

The terms ‘adaptive design’ and ‘adaptive trial’ were included as it was likely that the registry may say that an AD was/will be used without detailing the adaptations. The retrieved trials were subjected to review to determine whether the considered adaptations were relevant to RAR.

### 2.4 Selection process, data extraction and quality control

For the identified clinical trials, sections relating to study details were reviewed first to identify trial eligibility during the initial stage. If a trial was not excluded at this stage, a comprehensive review of the full text was undertaken, and accessible protocols, SAPs and related publications were retrieved. Trials from each search assessed as eligible were then combined, and duplicates were identified based on unique identifiers, such as clinical trial registration number(s). This sequential process allowed for a thorough assessment of the trial's relevance and eligibility before delving into more detailed data extraction. Reasons for exclusion were documented at the various stages.

Investigators and authors of trial records that required further information or clarification to address uncertainties were contacted whenever possible. This aimed to enhance the accuracy and completeness of the data extraction process by seeking additional details or clarifications. For example, it was unclear whether some published methods papers describing the design of RAR trials were purely hypothetical, real trials that were later conducted or trials in the set-up process.

One reviewer (IW) screened records for eligibility; an additional reviewer's (MD) opinion was sought where this was unclear. One reviewer (IW) extracted data from all trials. Simultaneously, an additional reviewer (MD) independently reviewed a subset of five eligible trials to check for the level of agreement in data extraction. These five trials were selected purposely to cover specific characteristics (e.g., platform versus non-platform, complexity of design). An excellent agreement, which ranged from 85.7% to 97.6%, as measured by the proportion of data items agreed on between the reviewers per record, was observed. Based on this, a further independent review was deemed unnecessary. IW and MD discussed and resolved any discrepancies in the extracted data.

Further quality checks ensured consistency between specific characteristics recorded during data extraction. For instance, when a trial was identified as a platform trial, one of the trial adaptations considered alongside RAR needed to be identified as adding arms. Some issues, such as how to handle multiple publications of results from a single platform, were discussed and resolved by all researchers. See the section ‘Dealing with platform trials’ for further details.

### 2.5 Data items

All authors contributed to the development of data items recorded during data extraction. The data items described in Supplementary Materials, Table 9 were collected for each trial. This involved various aspects, including general characteristics, trial design, operational characteristics, the RAR algorithm, decision-making criteria, trial results and any discussions and resources related to RAR. Certain fields required a degree of subjective judgment during the data extraction process, such as ‘Was what was carried out consistent with what was described at the design stage?’.

### 2.6 Dealing with platform trials

Platform trials posed unique challenges, prompting the decision, after discussion with all authors, to address them on a case-by-case basis. In broad terms, we categorised them into two types. The first type (e.g., NCT02977780) involved retrieving data from the master protocol, applicable when sub-trials within the platform utilised the same RAR methods, shared a common control arm, and had consistent underlying statistical goals or hypotheses. The second type (e.g., NCT05137119) involved extracting data from each trial within the platform but counting them collectively as a single RAR trial rather than counting each separately, as long as they utilised the same RAR methods. This approach was implemented when, at the very least, the control arm varied across the different trials.

A similar approach was taken for platform trial results (where available). Data were extracted from each results manuscript individually, thereby increasing the denominator when reporting results.

### 2.7 Data analysis and reporting

The results of this study were reported in line with the PRISMA guidance.^23^ The analysis was descriptive and narrative, without formal hypothesis testing. Continuous variables were summarised using mean and standard deviation (SD) or median and interquartile range (IQR) depending on the observed underlying distribution (i.e., whether it was skewed or normally distributed), as well as the minimum and maximum. Categorical variables were summarised using numbers and proportions relative to the appropriate denominator (e.g., eligible trials). Appropriate data visualisation methods were also used to present certain results. The main methods used in the RAR algorithms were mapped to classifications as described in most recent literature.^16^

Some variables/data items were estimated/calculated based on information from other variables. For example, when the number of interim analyses was not specified, it was approximated from the timing of interim analyses (e.g., periodically specified, such as weekly or monthly) and the specified trial duration. The proportion of sample size was calculated as the fraction of the number of participants at a given period of interest (e.g., at the burn-in period) relative to the maximum (or expected, if the maximum was not reported) number of participants. The sample size saving was calculated by the percentage change from the maximum (or expected, if the maximum was not reported) to the actual sample size.

## 3. Results

### 3.1 Trial selection

**Figure 3.**
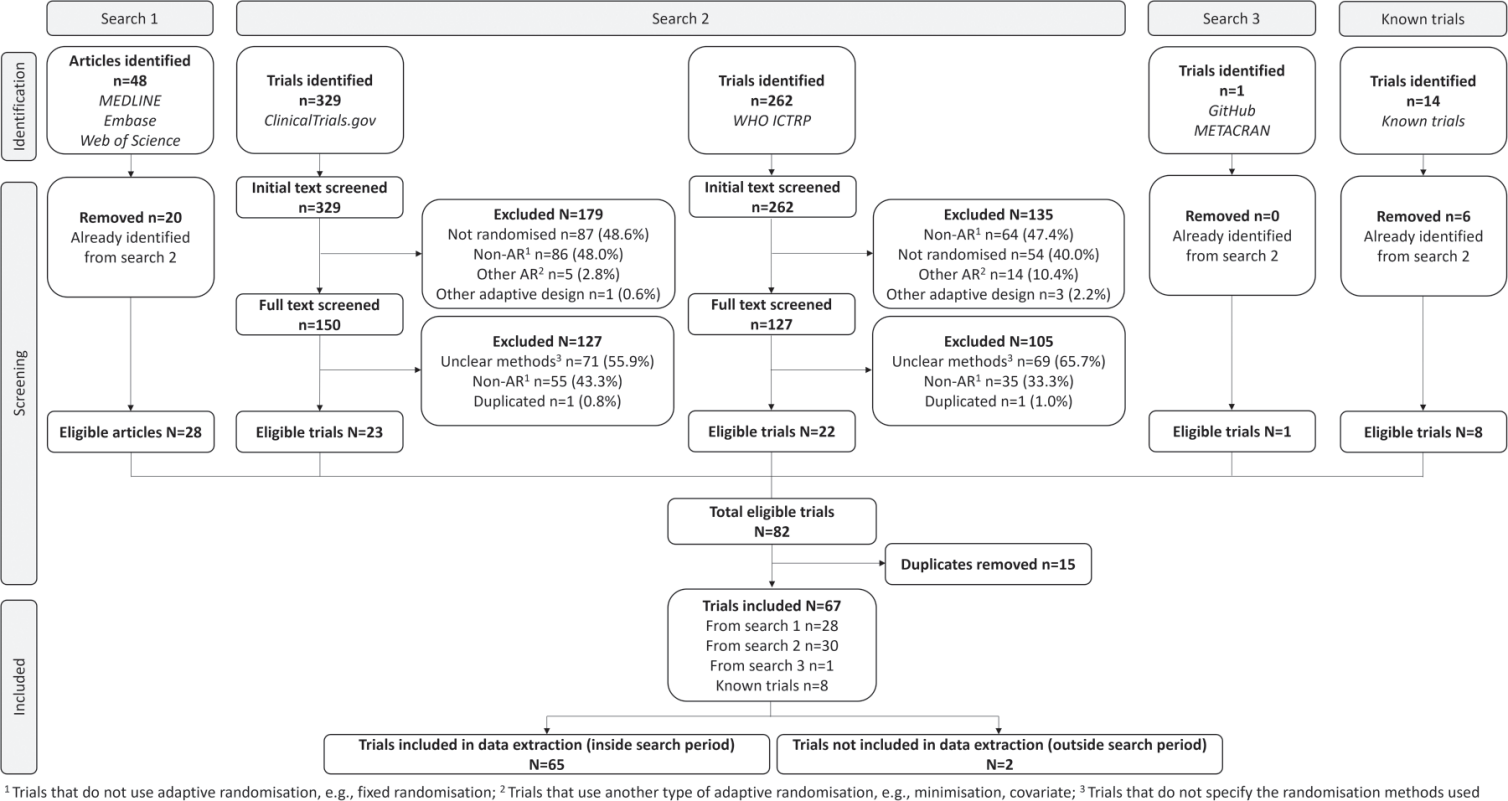
PRISMA flow diagram.

Figure 3 shows the flow of retrieved articles/trials from the four data sources. Initially, 591 trials were identified from search strategy 2. After screening, a total of 45 trials were eligible. Of the 48 articles identified from search strategy 1, 20 duplicates were removed as they had been identified from search strategy 2. Only one trial was identified from search strategy 3. By combining the three search strategies, eight trials known to researchers (fourth data source) and removing 15 duplicates, a total of 67 trials were identified. Two were excluded as they were outside the search period, leaving 65 eligible trials, which can be found in Supplementary Materials, Table 1.

### 3.2 Trends in the application of RAR in trials

**Figure 4.**
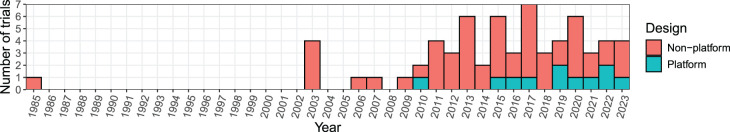
Number of RAR trials over the years.

Figure 4 gives the number of trials by year first reported, starting with the earliest, the ECMO trial^24^ in 1985. Subsequently, there was a notable eighteen-year gap before the occurrence of the following four trials in 2003. Overall, there was a slight increase in the number of trials from 1985 to 2023, reaching a peak in 2017. The first platform trial identified was in 2010 (NCT01042379).

### 3.3 Characterisation of general aspects

Table 1 presents the general characteristics of the 65 trials designed using RAR methods. As of October 2023, 34 (52.3%) trials had completed recruitment with available results. Further interim results were accessible in two of the 18 trials with ongoing recruitment.^25,26^

**Table 1.** General characteristics of the included trials.

	Total	Non-platform	Platform
Variable	*N* = 65	*N* = 54	*N* = 11
Recruitment status (as of October 2023)			
Completed	34 (52.3%)	33 (61.1%)	1 (9.1%)
In progress	18 (27.7%)	11 (20.4%)	7 (63.6%)
Not yet started	6 (9.2%)	5 (9.3%)	1 (9.1%)
Terminated	7 (10.8%)	5 (9.3%)	2 (18.2%)
Disease area			
Bleeding	2 (3.1%)	2 (3.7%)	0 (0.0%)
Bone	5 (7.7%)	5 (9.3%)	0 (0.0%)
Brain disorders	5 (7.7%)	5 (9.3%)	0 (0.0%)
Cardiovascular	2 (3.1%)	1 (1.9%)	1 (9.1%)
Infections	7 (10.8%)	6 (11.1%)	1 (9.1%)
Infectious disease	6 (9.2%)	3 (5.6%)	3 (27.3%)
Menstruation	2 (3.1%)	2 (3.7%)	0 (0.0%)
Mental health	4 (6.2%)	4 (7.4%)	0 (0.0%)
Oncology	16 (24.6%)	12 (22.2%)	4 (36.4%)
Ophthalmology	1 (1.5%)	1 (1.9%)	0 (0.0%)
Perinatal	1 (1.5%)	1 (1.9%)	0 (0.0%)
Respiratory	4 (6.2%)	3 (5.6%)	1 (9.1%)
Sciatica	1 (1.5%)	1 (1.9%)	0 (0.0%)
Sleep-related	2 (3.1%)	2 (3.7%)	0 (0.0%)
Stroke	5 (7.7%)	4 (7.4%)	1 (9.1%)
Urological	2 (3.1%)	2 (3.7%)	0 (0.0%)

Of the 59 trials that were completed, in progress or terminated, 20 (33.9%), 46 (78.0%) and 13 (22.0%) had accessible protocols and SAPs, protocols only and neither protocols nor SAPs, respectively. Of the 45 (69.2%) trials that did not have accessible standalone SAPs, 18 (40.0%) provided sufficient statistical detail related to RAR elsewhere, such as method articles or protocols.

#### 3.3.1 Research setting and disease area

The leading research setting that applied RAR was in oncology at nearly a quarter of trials, followed by infections (e.g., sepsis) and infectious diseases (e.g., COVID-19) (Table 1). However, RAR was applied in more diverse areas such as stroke, respiratory, bone, brain disorders and mental health.

#### 3.3.2 Geographical location of lead investigators and recruiting sites

**Figure 5.**
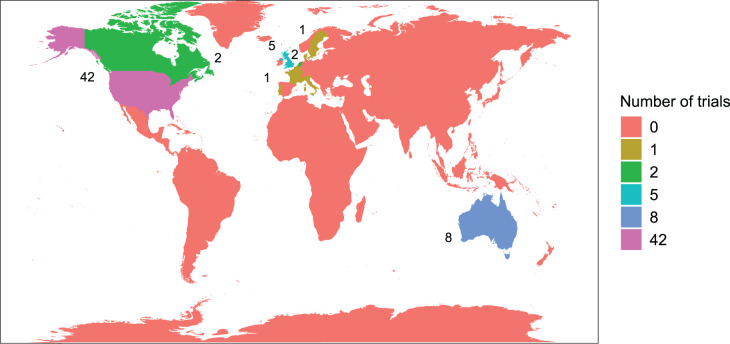
Location of lead investigator(s) by continent.

Lead investigators were based in 12 countries, mostly North America and Europe. The United States had by far the largest proportion at 63.1%, followed by Australia at 12.3% and the United Kingdom at 7.7%. Figure 5 displays a map of these results.

**Figure 6.**
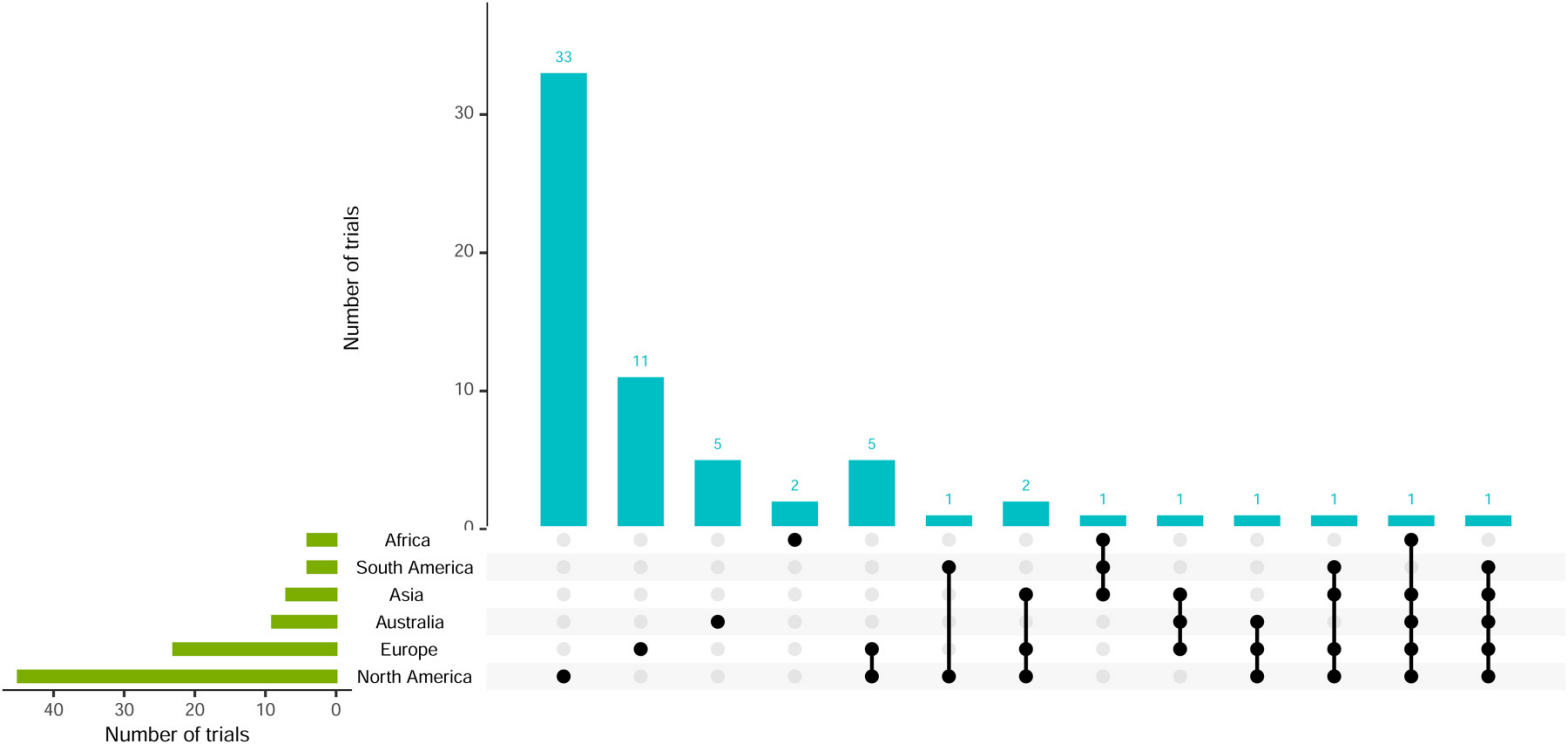
Visualisation of recruiting sites and combinations.

Although 51 (78.5%) trials were conducted in a single continent, other trials were conducted in sites across as many as five continents. Figure 6 shows the recruiting sites, showing a similar trend to the location of lead investigators. North America recruited the most sites (69.2%), followed by Europe (35.4%). Of the 11 platform trials, four recruited in North America, three in Europe and three in both.

### 3.4 Characterisation of trial design aspects

All trials applied RAR in the context of individual randomisation, with most having at least a phase II component (40, 61.5%) and parallel-group design (54, 83.1%) (Table 2). Of the four trials with ‘unclear’ details of the trial design, only one had a protocol available, whereas the other three had neither an accessible protocol nor SAP. There were 11 platform trials applied across trial phases, and four (36.4%) of these were factorial in nature.^25^ Bayesian methods were used in 84.6% of trials (at the design and/or analysis stage), with all platform trials exclusively employing them. Most trials were designed with superiority hypotheses (86.1%), while only^2^ platform trials had multiple hypotheses, and six (9.2%) had unclear hypotheses.

**Table 2.** Trial design characteristics of the included trials.

	Total	Non-platform	Platform
Variable	*N* = 65	*N* = 54	*N* = 11
Trial design			
Factorial	6 (9.2%)	2 (3.7%)	4 (36.4%)
Parallel group	54 (83.1%)	47 (87.0%)	7 (63.6%)
Umbrella	1 (1.5%)	1 (1.9%)	0 (0.0%)
Unclear	4 (6.2%)	4 (7.4%)	0 (0.0%)
Trial phase			
Phase 1/2	2 (3.1%)	1 (1.9%)	1 (9.1%)
Phase 2	32 (49.2%)	30 (55.6%)	2 (18.2%)
Phase 2/3	6 (9.2%)	5 (9.3%)	1 (9.1%)
Phase 3	12 (18.5%)	9 (16.7%)	3 (27.3%)
Phase 4	4 (6.2%)	2 (3.7%)	2 (18.2%)
Unclear	3 (4.6%)	2 (3.7%)	1 (9.1%)
Not applicable	6 (9.2%)	5 (9.3%)	1 (9.1%)
Nature of primary hypothesis			
Superiority	54 (83.1%)	46 (85.2%)	8 (72.7%)
Superiority + equivalence	1 (1.5%)	0 (0.0%)	1 (9.1%)
Superiority + non-inferiority	1 (1.5%)	0 (0.0%)	1 (9.1%)
Non-inferiority	3 (4.6%)	3 (5.6%)	0 (0.0%)
Unclear	6 (9.2%)	5 (9.3%)	1 (9.1%)
Nature of statistical framework			
Frequentist	9 (13.8%)	9 (16.7%)	0 (0.0%)
Bayesian	41 (63.1%)	30 (55.6%)	11 (100.0%)
Both	14 (21.5%)	14 (25.9%)	0 (0.0%)
Unclear	1 (1.5%)	1 (1.9%)	0 (0.0%)
Nature of blinding			
Blinded	34 (52.3%)	33 (61.1%)	1 (9.1%)
Unblinded	28 (43.1%)	19 (35.2%)	9 (81.8%)
Unclear	3 (4.6%)	2 (3.7%)	1 (9.1%)
Nature of treatment(s)			
Behavioural therapy	4 (6.2%)	4 (7.4%)	0 (0.0%)
Biological	1 (1.5%)	0 (0.0%)	1 (9.1%)
Biological + drug	1 (1.5%)	0 (0.0%)	1 (9.1%)
Clinical management	1 (1.5%)	1 (1.9%)	0 (0.0%)
Device	3 (4.6%)	2 (3.7%)	1 (9.1%)
Drug	49 (75.4%)	43 (79.6%)	6 (54.5%)
Drug + scan	1 (1.5%)	0 (0.0%)	1 (9.1%)
Drug + surgery	1 (1.5%)	1 (1.9%)	0 (0.0%)
Financial incentive	1 (1.5%)	1 (1.9%)	0 (0.0%)
Physiotherapy	2 (3.1%)	2 (3.7%)	0 (0.0%)
Surgery	1 (1.5%)	0 (0.0%)	1 (9.1%)
Nature of comparator(s)			
Active treatment	18 (27.7%)	14 (25.9%)	4 (36.4%)
No + active treatment	2 (3.1%)	0 (0.0%)	2 (18.2%)
No designated comparator	20 (30.8%)	19 (35.2%)	1 (9.1%)
No treatment	8 (12.3%)	4 (7.4%)	4 (36.4%)
Placebo	17 (26.2%)	17 (31.5%)	0 (0.0%)
Categorised number of arms			
2 arms	15 (23.1%)	15 (27.8%)	0 (0.0%)
>2 arms	41 (63.1%)	38 (70.4%)	3 (27.3%)
Ongoing platform	8 (12.3%)	0 (0.0%)	8 (72.7%)
Variable	1 (1.5%)	1 (1.9%)	0 (0.0%)
Number of arms	*n* = 56	*n* = 53	*n* = 3
Median (IQR)	4 (2, 6)	4 (2, 6)	4 (4, 6)
Min, Max	2, 16	2, 16	4, 7
Type of primary outcome(s)			
Binary	39 (54.9%)	32 (54.2%)	7 (58.3%)
Continuous	19 (26.8%)	19 (32.2%)	0 (0.0%)
Ordinal categorical	4 (5.6%)	3 (5.1%)	1 (8.3%)
Time-to-event	9 (12.7%)	5 (8.5%)	4 (33.3%)
Days to observe primary outcome(s)^a^	*n* = 61	*n* = 51	*n* = 10
Median (IQR)	56.0 (21.0, 90.0)	56.0 (14.0, 87.0)	90.0 (28.5, 148.5)
Min, Max	0, 1095	0, 730.5	14, 1095
Trial population			
Child	3 (4.6%)	3 (5.6%)	0 (0.0%)
Adult	2 (3.1%)	2 (3.7%)	0 (0.0%)
Older adult	4 (6.2%)	4 (7.4%)	0 (0.0%)
Child + adult	2 (3.1%)	2 (3.7%)	0 (0.0%)
Adult + older adult	45 (69.2%)	37 (68.5%)	8 (72.7%)
Child + adult + older adult	9 (13.8%)	6 (11.1%)	3 (27.3%)

^a^ Different denominator to rest of table due to the availability of information.

#### 3.4.1 Extent of blinding

**Figure 7.**
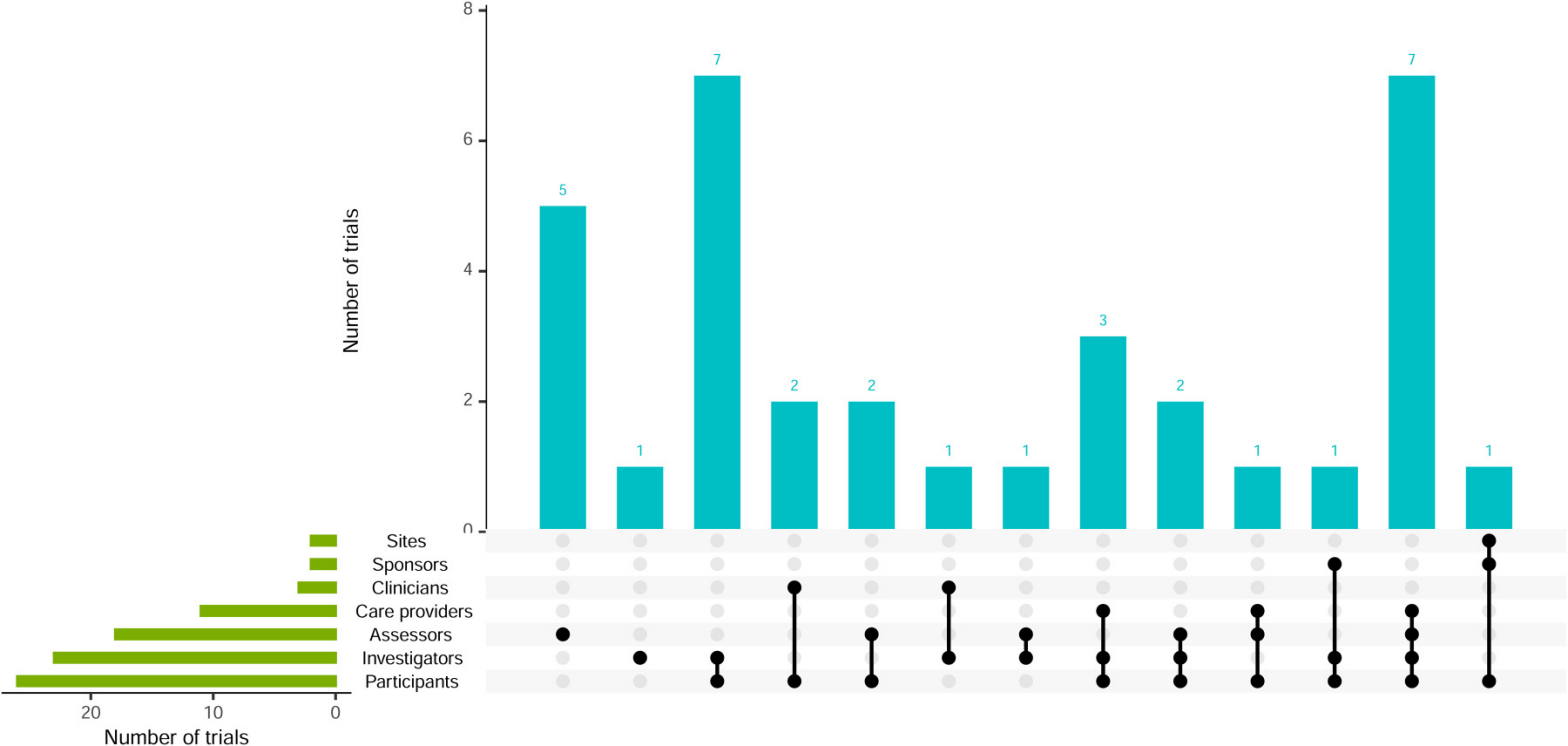
Visualisation of trial blinding and combinations.

Almost all platform trials had no blinding, except 1 that only blinded outcome assessors^27^ and another with unclear blinding (Table 2). A total of 34 (52.3%) trials had some form of blinding, with details shown in Figure 7. Participants, investigators and outcome assessors were blinded in 26 (76.5%), 23 (67.6%) and 18 (52.9%) trials, respectively. The combination of investigators and participants, or investigators, participants, assessors and care providers, were the most frequent.

#### 3.4.2 Nature of treatment and comparator arms

Of the trials, 52 (80%) focused on drug-related treatments, with the remainder examining other interventions such as behavioural therapy, devices and physiotherapy (Table 2). Only three trials (4.6%) investigated multiple treatment types, such as combining drugs and surgical procedures. Most trials included either no designated comparator, an active comparator or a placebo, with similar numbers across these comparator categories.

**Figure 8.**
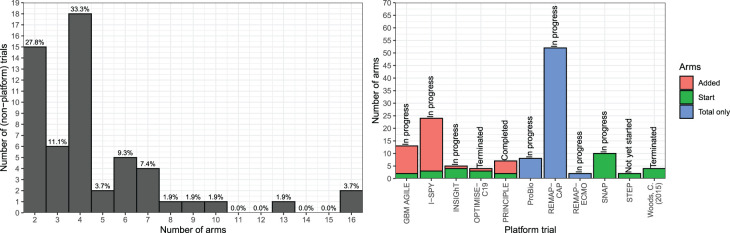
Distribution of the number of treatment arms, including comparator(s), in non-platform (left) and platform (right) trials.

It was possible to ascertain the number of treatment arms, including comparator(s), in 56 (86.2%) trials, which included three completed or terminated platform trials (Figure 8, left panel). Of these 56 trials, 41 (73.2%) had more than two treatment arms; the median (IQR) was 4 (2–6). One unique trial investigating financial incentives had a variable number of arms.^28^ It specified a range of incentive amounts, with the exact randomisation amounts determined by a computer algorithm as the trial progressed.

Of the 11 platform trials, eight were ongoing, so the exact number of treatment arms could not be established. More details about the status of these platform trials and the snapshot distribution of treatment arms, including those at the start of the trial and those that were added, are displayed in Figure 8 (right panel). It shows the current (subject to change as the trials progress) number of arms for each platform trial, split into starting number and number added. Where only the total number of arms is displayed (ProBio, REMAP-CAP, REMAP-ECMO), there was not enough available information to break it down into these two categories. Platform trials had a higher median number of arms at seven compared to non-platform trials at 4.

#### 3.4.3 Nature of primary outcomes

Thirty-nine (54.9%) trials used binary primary outcomes (Table 2). Most (90.8%) trials had one primary outcome, and only six trials had co-primary outcomes of different types (see Supplementary Materials, Figure 13). The majority of trials observed the primary outcome within 120 days (∼4 months), with a median (IQR) of 56 (21–90) days, and a wide range of 30 min to over three years. The trials with the longest time to observe the primary outcomes were likelier to have time-to-event outcomes.

### 3.5 Characterisation of RAR and other trial adaptations

#### 3.5.1 Application of RAR algorithms

Tables 3 and 4 detail features of the RAR algorithms. Most trials, 54 (83%), employed Bayesian RAR (BRAR). Trials using frequentist frameworks used a variety of algorithms. Still, they could mainly be categorised into the following two methods: two used design-driven and non-parametric, and five used optimal allocation targets. Of the four trials that did not provide an adequate description of the algorithm to be able to classify these; two failed to provide any information at all, and the other two provided minimal details, such as ‘adapted according to the dose–response curve seen at the interim analysis’ and ‘guided by the adaptive algorithm’, without describing how the RAR was to be carried out. Overall, 19 trials failed to provide clear information on the statistical data required, including the four uncategorised methods. Of the 54 trials that used BRAR, 78% gave sufficient statistical information, compared to four of the seven (57%) trials that used the other two methods.

**Table 3.** RAR algorithm classification details of the included trials.

		Design-driven and	Not enough	
		non-parametric	information	Optimal
Variable	*N* = 54	*N* = 2	*N* = 4	*N* = 5
Nature of statistical framework				
Bayesian	41 (75.9%)	0 (0.0%)	0 (0.0%)	0 (0.0%)
Both	13 (24.1%)	0 (0.0%)	0 (0.0%)	1 (20.0%)
Frequentist	0 (0.0%)	2 (100.0%)	3 (75.0%)	4 (80.0%)
Unclear	0 (0.0%)	0 (0.0%)	1 (25.0%)	0 (0.0%)
Adaptation outcome type				
Binary	31 (57.4%)	2 (100.0%)	2 (50.0%)	2 (40.0%)
Continuous	14 (25.9%)	0 (0.0%)	2 (50.0%)	2 (40.0%)
Ordinal categorical	2 (3.7%)	0 (0.0%)	0 (0.0%)	0 (0.0%)
Time-to-event	7 (13.0%)	0 (0.0%)	0 (0.0%)	1 (20.0%)

**Table 4.** RAR algorithm details of the included trials.

Variable	Summary (*N* = 65)
Group level applied at	
Overall treatment arm	49 (75.4%)
Subgroup/stratum within treatment arms	15 (23.1%)
No information	1 (1.5%)
Adjusted for covariates	
No	48 (73.8%)
Yes	11 (16.9%)
Unclear	6 (9.2%)
Incorporate safety data	
No	55 (84.6%)
Yes	4 (6.2%)
Unclear	6 (9.2%)
Allocation to arms restricted	
No	30 (46.2%)
Yes	33 (50.8%)
Unclear	2 (3.1%)
If yes to restriction, specify details^a^	*n* = 33
Control group at least the same as any single treatment arm	1 (1.5%)
Fixed control proportion	19 (29.2%)
Fixed control proportion + min and/or max proportions for each arm	1 (1.5%)
Fixed lowest dose proportion	1 (1.5%)
Proportion fixed for added arms until ≥1 events are observed in that arm	1 (1.5%)
Allocate to superior arm if subgroup meets superiority criterion	1 (1.5%)
Min and/or max proportions for each arm	7 (10.8%)
Unclear	2 (3.1%)
Employed a burn-in period	
No	4 (6.2%)
Yes	57 (87.7%)
Unclear	4 (6.2%)
If yes to burn-in, randomisation method used^a^	*n* = 57
Simple randomisation	16 (28.1%)
Blocked randomisation	8 (14.0%)
Stratified randomisation	4 (7.0%)
Stratified blocked randomisation	1 (1.8%)
No information	28 (49.1%)
If yes to burn-in, allocation ratio specified^a^	*n* = 57
No	5 (8.8%)
Yes	51 (89.5%)
No information	1 (1.8%)
If yes to burn-in, proportion of total sample size (%)^a^	*n* = 49
Mean (SD)	21.6 (11.8)
Median (IQR)	20.0 (13.3, 27.8)
Min, Max	2.7, 57.1

^a^ Different denominator to rest of table due to conditional responses.

All but one trial with a time-to-event adaptation outcome used BRAR. This trial used a time-to-event adaptation of the group sequential Doubly-adaptive Biased Coin Design.^29^ One trial used the randomised play-the-winner rule: a binary outcome, design-driven trial. Other common algorithms for frequentist designs were the multi-arm bandit, a computer algorithm determining the ‘optimal’ dose for minimising the expected variance of the response at the minimal dose, achieving near maximal efficacy, and minimal sufficient balancing (discussed further in the section ‘Real-life case studies’).

Fewer than 25% of trials applied algorithms at the subgroup/stratum level within arms, including 64% of platform trials. Most algorithms did not adjust for covariates or incorporate safety data. About half restricted allocation to certain arms, with the most common method fixing the control arm proportion. Some also set maximum and/or minimum allocation proportions for each arm.

Nearly all, 88%, of trials employed a burn-in period. However, just over half of these trials specified the randomisation method used for this. The reporting of allocation ratios was more comprehensive, with 89% specifying this aspect. The proportion of the sample size was available for 82% of trials, and there was a wide range of values for this, although all but one were under 50%. Of the 15 trials that employed the RAR algorithm at the subgroup/stratum level, all but one^30^ still applied the burn-in to the overall level.

In the SNAP trial,^27^ a platform trial with five domains, RAR would only be considered in domains with more than two interventions. As all the domains initially start with two arms, RAR will only be used if arms are added; if not, the randomisation will remain equal. This trial is still in progress (at the time of data extraction), so it is not yet clear if RAR will be activated in any domains.

#### 3.5.2 Other trial adaptations applied alongside RAR

**Figure 9.**
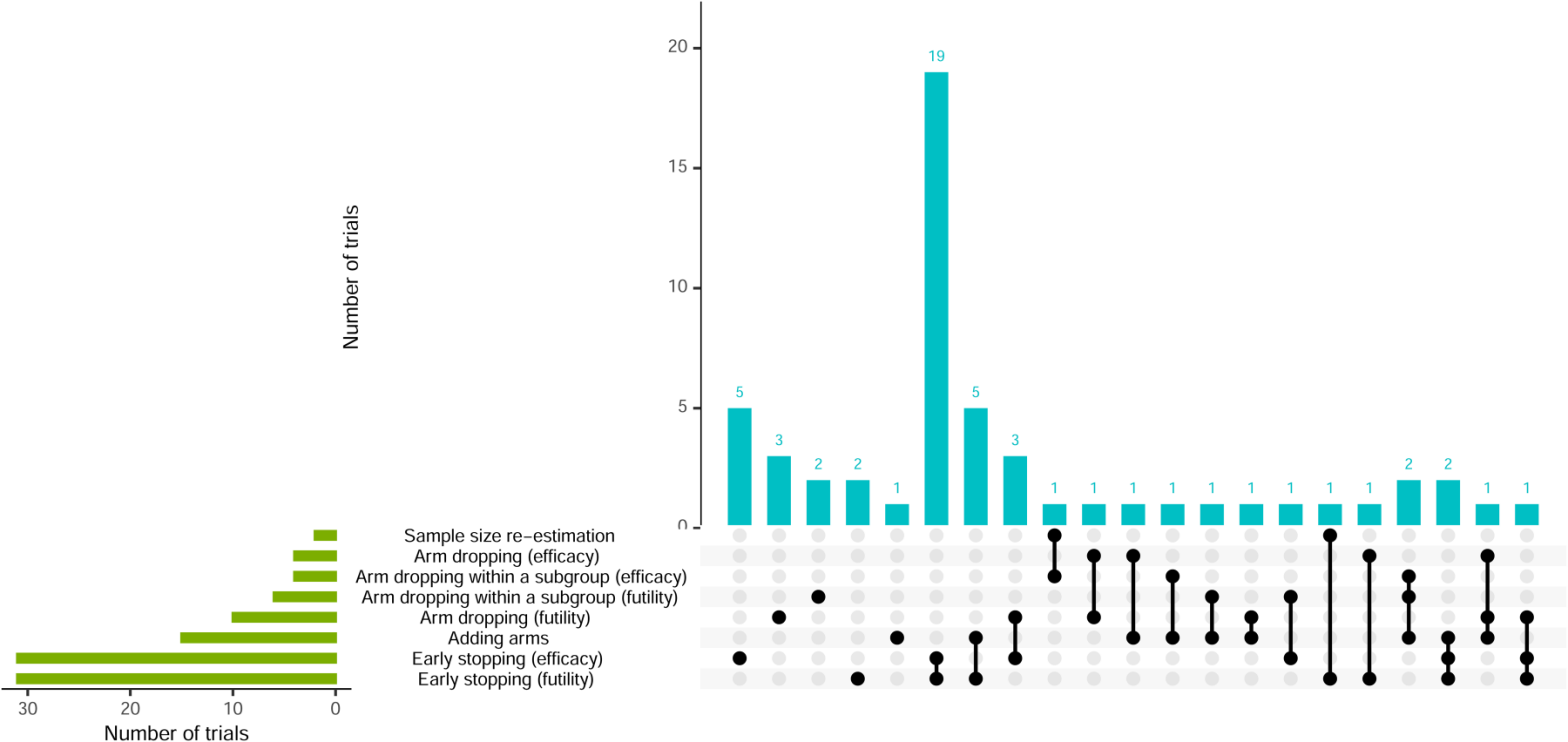
Visualisation of trial adaptations and combinations (alongside RAR).

Over 85% of trials had other adaptations in addition to RAR. Figure 9 shows the different combinations of adaptations, with 54% using two. Most of these employed early trial stopping for both efficacy and futility. Arm dropping for futility was more common than arm dropping for efficacy. Additionally, arm dropping for the whole trial population was used in 22% of trials, whereas arm dropping within specific subgroups of the trial (i.e., adaptive population enrichment) was used slightly less, at 15%.

#### 3.5.3 Interim analyses

The number of interim analyses was available for just over half of trials, including estimates for 13 (Table 5). Most trials had fewer than 20, with five being the most common. One extreme trial had approximately 91 interim analyses, estimated based on 24-h intervals and a 3-month duration.^31^ Confidentiality of interim data was poorly addressed, with 75% of trials failing to describe measures to minimise potential operational bias. However, about half provided better documentation of those responsible for interim decisions and recommendations.

**Table 5.** Interim analysis characteristics of the included trials.

Variable	Summary (*N* = 65)
Outcome used for interim analyses different from the primary	
No	61 (93.8%)
Yes	4 (6.2%)
Number and/or timing of interim analyses described	
Both	31 (47.7%)
Timing	15 (23.1%)
Neither	17 (26.2%)
Number	2 (3.1%)
Number of interim analyses^a^	*n* = 33
1	2 (3.1%)
2	3 (4.6%)
3–5	9 (13.8%)
6–10	6 (9.2%)
11–20	7 (10.8%)
≥21	6 (9.2%)
Clearly described confidentiality of interim data	
No	49 (75.4%)
Yes	16 (24.6%)
Clearly described who made interim recommendation/decisions	
No	34 (52.3%)
Yes	31 (47.7%)
Described methods for handling missing interim data	
No	40 (61.5%)
Yes	24 (36.9%)
Unclear	1 (1.5%)
Described approaches for handling missing interim data^a^	*n* = 24
Complete case	11 (45.8%)
Complete case + joint model	1 (4.2%)
Complete case + worst case	1 (4.2%)
Multiple + single imputation	1 (4.2%)
Multiple imputation	3 (12.5%)
Multiple imputation + worst case	1 (4.2%)
Other	2 (8.3%)
Single imputation	1 (4.2%)
Unclear	1 (4.2%)
Worst case	2 (8.3%)

^a^ Different denominator to rest of table due to conditional responses.

Only four trials used different outcome types for primary and interim analyses: in three, the outcome type was consistent, but interim outcomes were observed more quickly, while the fourth used a continuous variable for the primary outcome and a binary outcome for interim analysis, both assessed over the same 56-day period (Supplemental Materials, Table 11).

##### 3.5.3.1 Decision-making criteria

Most trials clearly described the nature of the decision-making criteria for other adaptations, with only 11% lacking this information. In two trials, the timing for implementing these criteria differed from RAR updates.^32,33^ For one trial, RAR and early stopping occurred bi-weekly, with RAR starting after 35 participants and early stopping after 150. In the other, early stopping began with 70 participants, and RAR with 14. Criteria for claiming evidence of the effect of treatment(s) were well described for 71% of trials. Of the nine trials where the criteria were not well described, eight had a protocol available. Of the remaining 10 trials where it was unclear if the criteria were well described, only two had a protocol available.

##### 3.5.3.2 Missing interim outcome data

Information on handling missing interim data was provided in 37% of trials (Table 5). One trial was classed as ‘unclear’ as the protocol stated that further details on handling missing data would be defined in the SAP, but the accessible SAP contained no related information. Most trials did not explicitly state ‘interim’ data but were classed as ‘yes’ if it implied that the methods applied to all data. Of the 24 trials with information, 13 (54.1%) used (at least) complete case analysis and seven (29.2%) used (at least) imputation approaches (single or multiple).

#### 3.5.4 Operating characteristics

The most frequently reported sample size was the expected, followed by maximum, with few providing minimum; and none reporting all three (Table 6). Most trials (69%) reported only one. Three trials failed to report any sample sizes: one was the ECMO trial,^24^ another stated in the protocol that ‘sample size is determined by case volume throughout the course of the pandemic’, and a platform trial stated there was ‘no pre-defined sample size’. The most common combinations of sample size were maximum and minimum, and maximum and expected. Over two-thirds of trials used simulations to determine sample size and operating characteristics. Also, 30/39 (77%) of trials that evaluated operating characteristics under different scenarios included scenarios under different treatment effects.

**Table 6.** Operating characteristics of the included trials.

Variable	Summary (*N* = 65)
Available sample sizes	
Maximum	33 (50.8%)
Minimum	10 (15.4%)
Expected	36 (55.4%)
Methods for determining sample size(s)	
Analytical methods	4 (6.2%)
Determined by case volume throughout the trial	1 (1.5%)
Simulations	42 (64.6%)
No information	18 (27.7%)
Methods for determining operating characteristics	
Simulations	43 (66.2%)
No information	22 (33.8%)
Probability of claiming specified hypothesis when treatment ineffective (e.g., type I error)	
No	30 (46.2%)
Yes	35 (53.8%)
Power specified	
No	24 (36.9%)
Yes	41 (63.1%)
Operating characteristics evaluated under different scenarios	
No	3 (4.6%)
Yes	39 (60.0%)
Unclear	23 (35.4%)
If yes, operating characteristics evaluated under different treatment effects^a^	*n* = 39
No	7 (17.9%)
Yes	30 (76.9%)
Unclear	2 (5.1%)
Priors described and justified (if applicable)^a^	*n* = 55
Yes	3 (5.5%)
Described only	13 (23.6%)
No	6 (10.9%)
Unclear	33 (60%)

^a^ Different denominator to rest of table due to conditional responses.

Reporting probability of treatment decision-making errors (e.g., power and type I error) was generally inadequate, with 35% of trials reporting neither of these. If one was reported, the other was reported in 81% of cases. Trials that used both Bayesian and frequentist methods had the highest proportion (79%) of reporting type I error. In over half of Bayesian method trials, priors were often ambiguous, yet their descriptions were more detailed than the justifications provided.

### 3.6 Characterisation of accessible trial results

**Figure 10.**
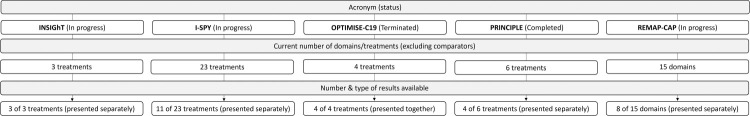
Flow diagram for included platform trials (with some/all results available).

Figure 10 describes the number and type (one manuscript versus multiple manuscripts) of results presented in relation to the number of treatments/domains for the platform trials. Table 7 shows the results of the 41 trials (37 non-platform and 5 platform) that had some form of results available.

**Table 7.** Results of the included trials.

	Total	Non-platform	Platform
Variable	*N* = 64^a^	*N* = 37	*N* = 27^a^
Nature of results presented			
Final	61 (95.3%)	37 (100.0%)	24 (88.9%)
Interim	3 (4.7%)	0 (0.0%)	3 (11.1%)
Was RAR actually used?			
No	5 (7.8%)	2 (5.4%)	3 (11.1%)
Yes	59 (92.2%)	35 (94.6%)	24 (88.9%)
Baseline data presented at each interim analysis			
No	64 (100.0%)	37 (100.0%)	27 (100.0%)
Interims conducted at specified time			
Yes	15 (23.4%)	13 (35.1%)	2 (7.4%)
Unclear	47 (73.4%)	23 (62.2%)	24 (88.9%)
Not applicable	2 (3.1%)	1 (2.7%)	1 (3.7%)
Allocation ratios reported over time			
No	32 (50.0%)	25 (67.6%)	7 (25.9%)
Yes	14 (21.9%)	11 (29.7%)	3 (11.1%)
Unclear	14 (21.9%)	0 (0.0%)	14 (51.9%)
Not applicable	4 (6.2%)	1 (2.7%)	3 (11.1%)
Other trial adaptations reported over time			
No	32 (50.0%)	25 (67.6%)	7 (25.9%)
Yes	9 (14.1%)	4 (10.8%)	5 (18.5%)
Unclear	14 (21.9%)	0 (0.0%)	14 (51.9%)
Not applicable	9 (14.1%)	8 (21.6%)	1 (3.7%)
Time trends accounted for in analysis			
No	53 (82.8%)	37 (100.0%)	16 (59.3%)
Yes	8 (12.5%)	0 (0.0%)	8 (29.6%)
Unclear	3 (4.7%)	0 (0.0%)	3 (11.1%)
Was implementation consistent with design plan?			
No	1 (1.6%)	1 (2.7%)	0 (0.0%)
Yes	39 (60.9%)	16 (43.2%)	23 (85.2%)
Unclear	24 (37.5%)	20 (54.1%)	4 (14.8%)
Decision-making criteria for claiming evidence at trial's end done as described			
Yes	52 (81.2%)	26 (70.3%)	26 (96.3%)
Unclear	12 (18.8%)	11 (29.7%)	1 (3.7%)
Decision-making criteria for trial adaptations done as described			
Yes	40 (62.5%)	17 (45.9%)	23 (85.2%)
Unclear	16 (25.0%)	12 (32.4%)	4 (14.8%)
Not applicable	8 (12.5%)	8 (21.6%)	0 (0.0%)
Actual sample size	*n* = 64	*n* = 37	*n* = 27
Mean (SD)	718.4 (1138.5)	377.5 (443.2)	1185.6 (1573.8)
Median (IQR)	244.5 (98.5, 797.8)	250.0 (122.0, 384.0)	144.0 (95.5, 2116)
Min, Max	4, 4997	4, 2244	9, 4997
Sample size saving (%)^b^	*n* = 36	*n* = 36	*n* = 0
Mean (SD)	21.5 (25.7)	21.5 (25.7)	-
Median (IQR)	16.8 (−0.1, 37.0)	16.8 (−0.1, 37.0)	-
Min, Max	−6.8, 92	−6.8, 92	-
Early stopping
Did not stop early	25 (39.1%)	23 (62.2%)	2 (7.4%)
Driven by trial adaptation	22 (34.4%)	10 (27.0%)	12 (44.4%)
Not driven by trial adaptation	9 (14.1%)	2 (5.4%)	7 (25.9%)
Unclear if stopped early	8 (12.5%)	2 (5.4%)	6 (22.2%)
Proportion allocated to treatment arms deemed to have highest efficacy compared to lowest			
Greater	14 (21.9%)	13 (35.1%)	1 (3.7%)
Unclear	3 (4.7%)	3 (8.1%)	0 (0.0%)
Not applicable	47 (73.4%)	21 (56.8%)	26 (96.3%)

^a^ From the 5 platform trials that had available results, there were 27 papers identified that compared different arms within these trials (see Figure 10 for further details).

^b^ Different denominator to rest of table due to the availability of information (i.e. did not provide maximum or expected sample size to allow calculation).

Two non-platform trials that did not end up implementing RAR stopped early: one for futility before reaching the second phase, and the other faced recruitment challenges due to a rare condition. One platform trial with three domains also halted early without using RAR, citing loss of equipoise, safety concerns and futility adaptations. Overall, 61% of all trials stopped early, with a higher rate of 93% for platform trials. Early stopping was often due to trial adaptations, though nine trials halted for other reasons, including safety and recruitment issues.

The timing of the actual interim analyses was often unclear, complicating comparisons to the design stage. Two trials were classified as ‘not applicable’: the first, a domain within a platform, was terminated before any interim analyses, while the second stopped early for efficacy before the first interim analysis relating to RAR. Changes in allocation ratio(s) and other adaptations throughout the trials were inadequately reported, though the former was slightly better addressed. The four trials classed as ‘not applicable’ did not trigger RAR (or other trial adaptations). Another was classed as ‘unclear’ as only the abstract for interim results was found.

#### 3.6.1 Benefits associated with RAR

**Figure 11.**
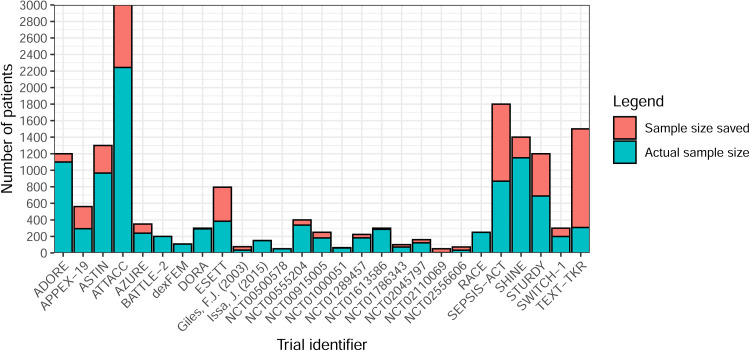
Sample size saving by (non-platform) trial (*n* = 37).

**Figure 12.**
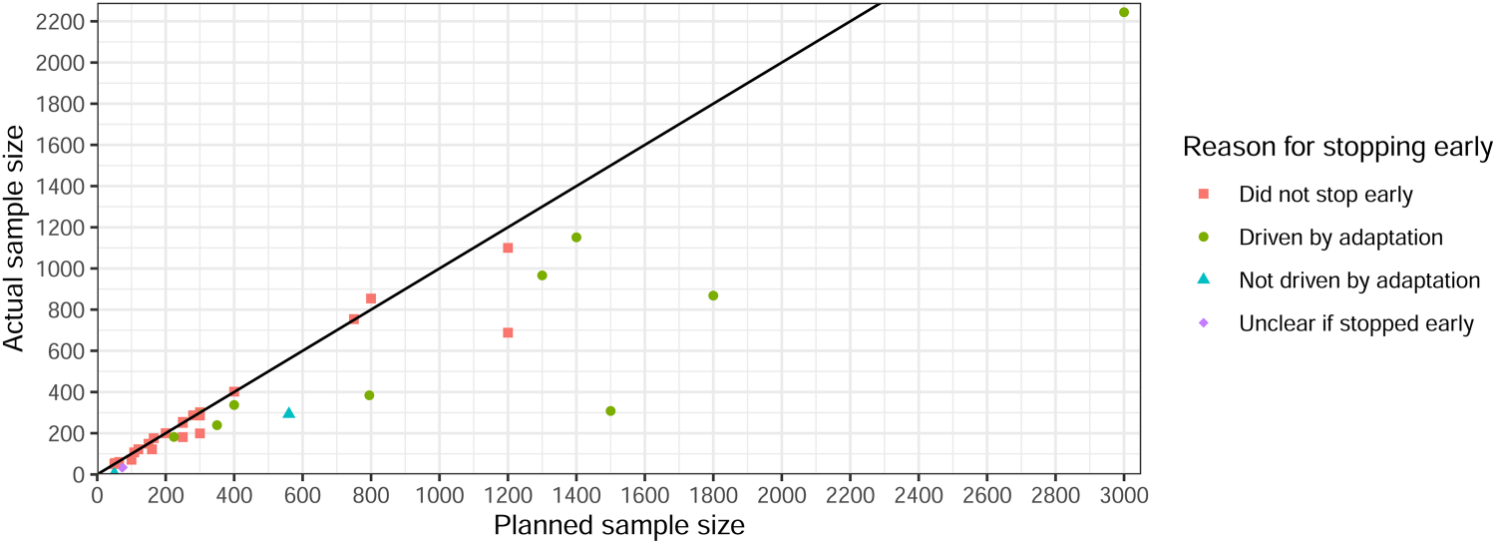
Planned versus actual sample size for non-platform trials (*n* = 27, excludes negative savings).

Sample size savings were calculated for 21 trials using the maximum sample size and 15 using the expected (Figures 11 and 12). In non-platform trials, only one trial had no maximum or expected sample size available, so the sample size saving could not be calculated. It was difficult to calculate savings for platform trials due to the nature of how results were presented (i.e., some presented separately and others together). Nine had a negative saving (actual sample size exceeded maximum/expected). Seven of these were based on the expected sample size, while two that used the maximum reported minimal increases of just two participants (400 vs. 402 and 300 vs. 302). One trial had a 92% saving but was terminated due to a rare condition and only recruited four participants.^34^

No trials recruited a higher proportion of participants to the arm(s) deemed to have the lowest efficacy. However, 5% were classed as ‘unclear’ due to issues finding the full manuscripts. Additionally, 73% were ‘not applicable’ for various reasons, such as no arms being efficacious or stopping early for futility.

### 3.7 Concerns discussed about the use of RAR

Several trials highlighted the need for interim outcome data to be quickly observed relative to recruitment rate.^20^ For instance, one trial with a time-to-event primary outcome reported a median observation time of around five months, delaying RAR updates. However, this trial's burn-in phase required recruiting 20 patients and observing one event in each group before updating allocations. One potential solution to address delayed RAR updates is the use of surrogate endpoints,^16^ as seen in four trials.^26,30,35,36^ However, one trial highlighted concerns about relying on an unvalidated interim or surrogate endpoint for trial adaptations. Another trial used an early surrogate endpoint for both primary and interim outcomes but noted that the relationship between this surrogate and the later clinically relevant endpoint had previously been questioned.^37^

Several trials raised concerns about unequal sample sizes between treatment arms, noting that this can reduce statistical power. As mentioned in the section ‘Application of RAR algorithms’, many trials addressed this by restricting allocation. However, one trial still experienced imbalances in key prognostic covariates between treatment arms.^38^ This could have occurred by chance due to the small sample size, with only 34 randomised participants. The authors acknowledged this limitation as an inherent possibility in any adaptive design and is not specific to RAR. It is also important to highlight that observed imbalances may not reduce the validity of trial results,^39^ but could impact trial credibility.^40^ It should be noted that this trial did not incorporate a burn-in period across arms before activating RAR, which most trials in this review did, and this could have lessened the problem.

Other identified issues include the resources required for RAR, the challenge of providing a simple explanation in the patient information sheet, and the provision of unblinded treatments that may allow clinicians and research teams to infer RAR proportions and interim treatment efficacy, which raises potential biases in certain adaptive designs including RAR (e.g., multi-arm multi-stage) in the fully unblinded setting. The controversies surrounding the ECMO trial^24^ have been discussed previously. For example, see Rosenberger and Lachin^41^ and Wilson et al.^20^ The main issue was withholding the intervention from the control patient, who subsequently died.

#### 3.7.1 Time trends

A common concern in the literature is time trends,^16,42^ discussed in multiple trials but largely unaccounted for (e.g., in analysis). Although it was often stated that possible bias would be introduced by changes in the study population over time.

Time trends were not accounted for in any of the trials that had completed recruitment (Table 7). However, three trials explicitly described the methods used to account for this, all were platform designs. One platform trial, REMAP-CAP,^43^ was still in progress at the time of data extraction and specified that the model would account for time trends by adjusting for 13-week time blocks. The STEP platform trial^44^ proposed an approach that achieved high statistical power and good patient benefit, in addition to being robust against population drift. It was motivated by the work of REMAP-CAP and incorporated a drift parameter to capture the change in treatment response rates in a modified Bayesian hierarchical drift model.^45^ The final platform trial^46^ was not initiated due to a decline in cases of the disease. However, it described in the protocol how population drift would be accounted for. It would use a prospective model that treated each month as a covariate and estimated the drift over time in the mortality rate (primary outcome).^12^

### 3.8 Statistical resources

Many trials made use of the Fixed and Adaptive Clinical Trial Simulator (FACTS)^47^ although it was often not described exactly what it was used for. However, FACTS has many capabilities, including trial simulation, flexible specification of burn-in periods, ability to fix control allocation proportions, setting allocation to 0 if a minimum RAR proportion is reached, controlling how aggressive the RAR is and use within platform designs (continuous and binary endpoints and Bayesian and frequentist analyses).

One trial, which had not yet started, stated in the protocol that details of the specific implementation of the multi-arm bandit algorithm would be available on request once the study is completed.^48^ No other code, packages or software were identified.

### 3.9 Real-life case studies

This section highlights some case study trials and exemplars. Table 8 details the trials, with a brief overview of the design, and the reason it has been highlighted. Note that INCEPT,^49^ was outside the search window (i.e., did not undergo data extraction) but is included as a good exemplar.

**Table 8.** Case studies.

Trial	Design	Details
INCEPT^49^	Platform	Various methodology papers, simulation studies and development of R package (adaptr). Clear website,^49^ with links to publications.
REMAP-CAP^43^	Platform	Clear website,^50^ with links to publications, protocols and SAPs (including for domains). Accounts for time-trends, detailed in SAP.
SAFER^51^	Non-platform	Automated trial, with daily interim analyses. Every 24 h, for 3 months, the probability of each arm being the best was estimated, using Monte-Carlo simulations to get posterior probability estimates.
SHINE^52^	Non-platform	One of the few frequentist RAR methods (minimal sufficient balancing design), which is well described in an additional paper.^53^
STEP^44^	Platform	Uses a unique and innovative RAR method, RARCOMP.^54^ A trade-off between RAR and fixed 1:1 allocation (where the allocation rate for treatment k is then the average of the RAR probability and the fixed).

## 4. Discussion

### 4.1 Key findings

Even though the use of RAR in clinical trials, particularly in platform designs, has increased since its first use in 1985, it still remains disproportionately low compared to the amount of methodological literature.^5–7^ North America dominates the field regarding lead investigators and recruiting sites, partly driven by companies such as Berry Consultants and the MD Anderson Cancer Center.

It is clear that RAR is still favoured in emergency care settings, with infections and infectious diseases, such as sepsis, COVID-19 and Ebola, making up a large proportion of the identified areas. However, oncology was the most common area, playing into RAR's advantages in severe medical conditions. A higher proportion of trials in these areas investigated drug-related treatments, potentially highlighting a specific context where RAR may be useful. RAR does not appear to limit the number of arms in a trial; rather, it was implemented across a wide range, including those with high numbers in platform trial designs. This suggests that RAR remains versatile, even though it is often considered particularly efficient in multi-arm trials according to the literature.^55,56^

Although continuous outcomes have generated more theoretical interest in recent years,^56–58^ binary outcomes still seem favoured, with time-to-event and categorical falling behind. The potentially long time to observe time-to-event outcomes and the lack of methodological literature on categorical outcomes might be the cause of this. We refer the reader to literature on RAR methods for time-to-event outcomes^59,60^ and categorical outcomes.^61,62^ Much literature discusses the need for the interim outcome data to be observed sufficiently quickly relative to recruitment.^63^ This seems to be the case in practice, with most trials observing the interim outcome within four months.

Despite abundant RAR literature for frequentist frameworks,^64,65^ relatively few seem to have been applied in practice. This could be due to existing controversies surrounding the RAR's application in this context, as discussed in the literature.^20^ In contrast, BRAR was found to be used for binary, continuous, (ordinal) categorical and time-to-event outcomes, highlighting its wide application in real trials.

#### 4.1.1 Realised benefits relating to the use of RAR

One of the main benefits of RAR is the ability to assign more participants to effective treatments.^16^ The identified trials show that this is achieved in practice as none allocated more participants to the least effective treatments. This is strengthened by the sample size savings found, particularly when early stopping was used as an additional trial adaptation, further reducing the number of participants on less effective treatments and allowing trials to answer important clinical questions more quickly. Hence, the benefits of RAR may be more pronounced when used in combination with other adaptations.

The complexity of setting up and carrying out RAR can put unnecessary and unwanted burdens on trial teams and funders.^41,66,67^ The cost, time and effort needed to do this are significant disadvantages. It was found that most identified trials used complex simulations to estimate operating characteristics and sample sizes, as well as the need to develop and program the RAR algorithm from scratch, as very few validated and reliable statistical resources are readily available. However, a wide range of interim analyses were used, with some doing as few as one, and most trials implementing other adaptations at the same time as RAR. This shows that the benefits can still be achieved with relatively little added work.

#### 4.1.2 Maintaining trial validity and integrity

It is the goal of every trial to provide reliable estimates of treatment effects for evaluating benefits and risks to reach the correct conclusions.^68^ Adaptive trials, including those that use RAR, require additional safeguards to ensure both the confidentiality of interim data and that interim analyses to inform trial adaptations and related decision-making processes do not introduce operational biases that could harm the credibility of trial results to inform practice. These safeguards have been widely discussed both in the literature,^3,69^ and the PANDA toolkit.^40^ Of note, some RAR trials have used fully automated procedures to maintain the confidentiality of interim data.^51^

Obtaining reliable estimates of treatment effects following RAR trials is challenging and an ongoing methodological problem.^16^ Currently, researchers tend to use conventional statistical methods for non-adaptive trials to analyse RAR trials.^70^ Existing statistical methods and resources for performing inference following trials using different types of adaptive designs have been summarised^71^ and are continuously updated via PANDA.^40^

Where possible, the blinding or masking of treatment allocation to different parties involved in the conduct of the trial and decision-making is essential to reduce the introduction of several biases and may be particularly important in RAR trials. The use of RAR in unblinded trial settings has the potential to allow caregivers, researchers, funders/sponsors, patients and the public to make inferred conclusions on the emerging treatment effect, which, in turn, could introduce operational bias. This problem can also occur in other adaptive designs such as MAMS and adaptive platform trials. It raises questions on the minimum level of blinding required depending on the context to minimise potential biases and maximise the value of these adaptive designs.

The concern raised by some researchers regarding unequal sample sizes warrants further consideration. A common misconception is that equal allocation always maximises statistical power.^72^ However, this only holds when treatment arms have equal variance - a condition rarely met - particularly for binary outcomes under the alternative hypothesis.^16^ Moreover, some trials are designed with unequal allocation to achieve set objectives other than maximising power.^73^ For example, when a control arm represents a well-characterised standard of care while an investigative treatment is relatively new, a larger allocation to the new arm may be preferred to get more statistical information on potential harms. In fact, in certain settings, unequal allocation can enhance power compared to equal allocation. Specifically, RAR algorithms are designed to achieve set trial objective(s).^16^ For example, optimal RAR algorithms, such as those employing Neyman allocation, explicitly target allocation ratios that maximise power by accounting for variance differences between treatment groups.^74^

#### 4.1.3 Gaps in reporting

Several aspects were identified that could improve the reporting of RAR trials, with many outlined in the Adaptive designs CONSORT Extension (ACE).^68^ Similar issues with inadequate reporting have been observed in other research on advanced ADs across all trial phases, where poor reporting has been highlighted as a widespread problem.^22,75,76^ Addressing these reporting challenges will ultimately strengthen the overall quality and impact of research in RAR trials.

Notably, several aspects could be described better to allow the reproducibility of carrying out the RAR algorithm. For example, there was poor reporting of statistical information required to employ the algorithms, including the randomisation method (e.g., simple, blocked) used for the burn-in period. Missing data methods, which are vital for RAR due to the high chance of this occurring while data are accruing, were also poorly reported, and it was often not clear if the methods applied to interim outcome data (i.e., for the RAR stage) or final outcome data (i.e., for the final analysis stage). The priors used were often poorly described and justified when using Bayesian RAR.

More general aspects that could be better reported include sample size,^31^ as most trials only pre-specified one (out of expected, maximum and minimum); and power and type I error, although this is less necessary for Bayesian trials. The number of interim analyses was poorly reported in the protocol and/or SAP, contributing to the lack of clarity in reporting when the interim analyses actually took place in the results. Measures to safeguard the confidentiality of interim information and descriptions of individuals responsible for interim decisions were poorly reported. No trials reported baseline data at each interim analysis, which is important to consider as the trial population could vary over time and potentially get worse (e.g., if participants with a worse baseline disease level join the trial later). Half of the trials (with results) did not report the changes in the allocation ratios and other adaptations over time. At the very least, the change in allocation ratios should be reported to show how the RAR algorithm allocated participants and to highlight any points of interest (e.g., if it was a lot higher after one interim analysis compared to the rest).

Enhancing the transparency and completeness of reporting in such trials is essential to improving the interpretability of results and reproducibility of methods, results and inferences.

### 4.2 Strengths and limitations

This review is the most comprehensive to date, examining many real-world trials that employed RAR and their associated design features and results. The inclusion of eight different data sources, including two major clinical trial registries, ensured a robust identification of trials using RAR methods. This broad scope provides valuable insights into the current state of RAR in practice and lays a strong foundation for future research and improvements in trial design, conduct, analysis and reporting.

Interim results were only considered if a trial was still in progress or if final results were unavailable, with only two trials meeting this criterion. Access to more interim results, even with final results available, would have been useful for comparing reporting practices. This could include assessing if trials focus on other outcomes, (e.g., safety over efficacy); whether they report current allocation ratios or allocation ratios up to that point; and how they report the primary outcome.

A practical decision was made not to conduct 100% quality control on the data extraction. However, based on the five trials reviewed in parallel (as discussed in the section ‘Selection process, data extraction and quality control’), this was deemed unnecessary. Nonetheless, some minimal, missed or incorrect data are likely to exist. While some additional details, such as characterising who exactly was responsible for making interim decisions - could have added further depth, the overall dataset was highly comprehensive and provides a strong framework for robust analysis and meaningful insights.

### 4.3 Future work

A parallel piece of work is ongoing that aims to review the statistical literature of RAR designs with immediate application in clinical trials comparing the efficacy or effectiveness of treatments. Briefly, it will describe proposed RAR algorithms, and aims to characterise the performance and context of these algorithms including their objectives. The results described in this paper will be compared to this parallel work to assess if what is described in the statistical literature is being translated into practice.

A second parallel piece of work aims to identify and characterise existing statistical software, packages or code that can be used to implement RAR methods, providing a valuable resource for trialists interested in implementing RAR in trials. Building on this, validating these already available statistical resources (especially in R and other open-source code/packages) would be valuable in seeing what could be improved and added. This will highlight gaps in the current resources that could be built upon. In particular, simulations were found to be commonly used in trials using RAR, so creating more resources to simulate sample sizes and operating characteristics (choice of burn-in, performance metrics, power, type I error, etc.) would help encourage the use of RAR, where suitable, in practice.

By addressing these gaps and expanding methodological and practical resources, we can enhance understanding of RAR, driving its wider adoption in trials where it has the potential to offer meaningful benefits.

### 4.4 Future directions

As part of this work, several contexts lacking RAR application were identified. This includes different statistical hypotheses (non-inferiority and equivalence); categorical and time-to-event outcomes; application at the subgroup level rather than the overall population; integration with cluster randomisation; frequentist algorithms; and designs beyond parallel groups, such as factorial and umbrella. The use of co-primary outcomes is an interesting area for future methodological research and application in real trials. There is limited literature,^77–79^ with most focusing on multiple binary outcomes or short and long-term outcomes.

Furthermore, future efforts could explore the ethical and practical considerations of disclosing RAR details during ongoing trials. While some protocols and SAPs specify that full algorithm details and code will be shared post-trial, it is worth investigating the extent to which interim results, like current allocation ratios, should be made public. Given the nature of RAR, even partial disclosure could signal which arms are showing greater benefit, raising questions about confidentiality and trial integrity. This is a vital area for qualitative research, as it will shape how RAR is communicated and implemented in future trials.

## 5. Conclusion

RAR has shown promise in clinical trials, particularly in severe medical conditions like sepsis, COVID-19 and oncology. It offers advantages such as assigning more participants to effective treatments, and saving time and resources. Despite growing use, RAR remains underutilised due to its complexity, cost and the need for significant simulations. This study, the most comprehensive review to date, highlights RAR's potential and identifies gaps in its application, particularly in non-inferiority hypotheses, time-to-event outcomes and algorithms at the subgroup level. Future efforts should focus on improving statistical tools and addressing ethical concerns to enhance the broader adoption and effectiveness of RAR in clinical research.

## Supplemental Material

sj-pdf-1-smm-10.1177_09622802251348183 - Supplemental material for Response adaptive randomisation in clinical trials: Current practice, gaps and future directions Supplemental material, sj-pdf-1-smm-10.1177_09622802251348183 for Response adaptive randomisation in clinical trials: Current practice, gaps and future directions by Isabelle Wilson, Steven Julious, Christina Yap, Susan Todd and Munyaradzi Dimairo in Medical Research

sj-pdf-2-smm-10.1177_09622802251348183 - Supplemental material for Response adaptive randomisation in clinical trials: Current practice, gaps and future directions Supplemental material, sj-pdf-2-smm-10.1177_09622802251348183 for Response adaptive randomisation in clinical trials: Current practice, gaps and future directions by Isabelle Wilson, Steven Julious, Christina Yap, Susan Todd and Munyaradzi Dimairo in Medical Research
