# Supplementary figures and images for: Response adaptive randomisation in clinical trials: Current practice, gaps and future directions
Source: Stat Methods Med Res. 2025 Jun 18;34(9):1851–74. doi: 10.1177/09622802251348183 (PMC12460923; doi:10.1177/09622802251348183)

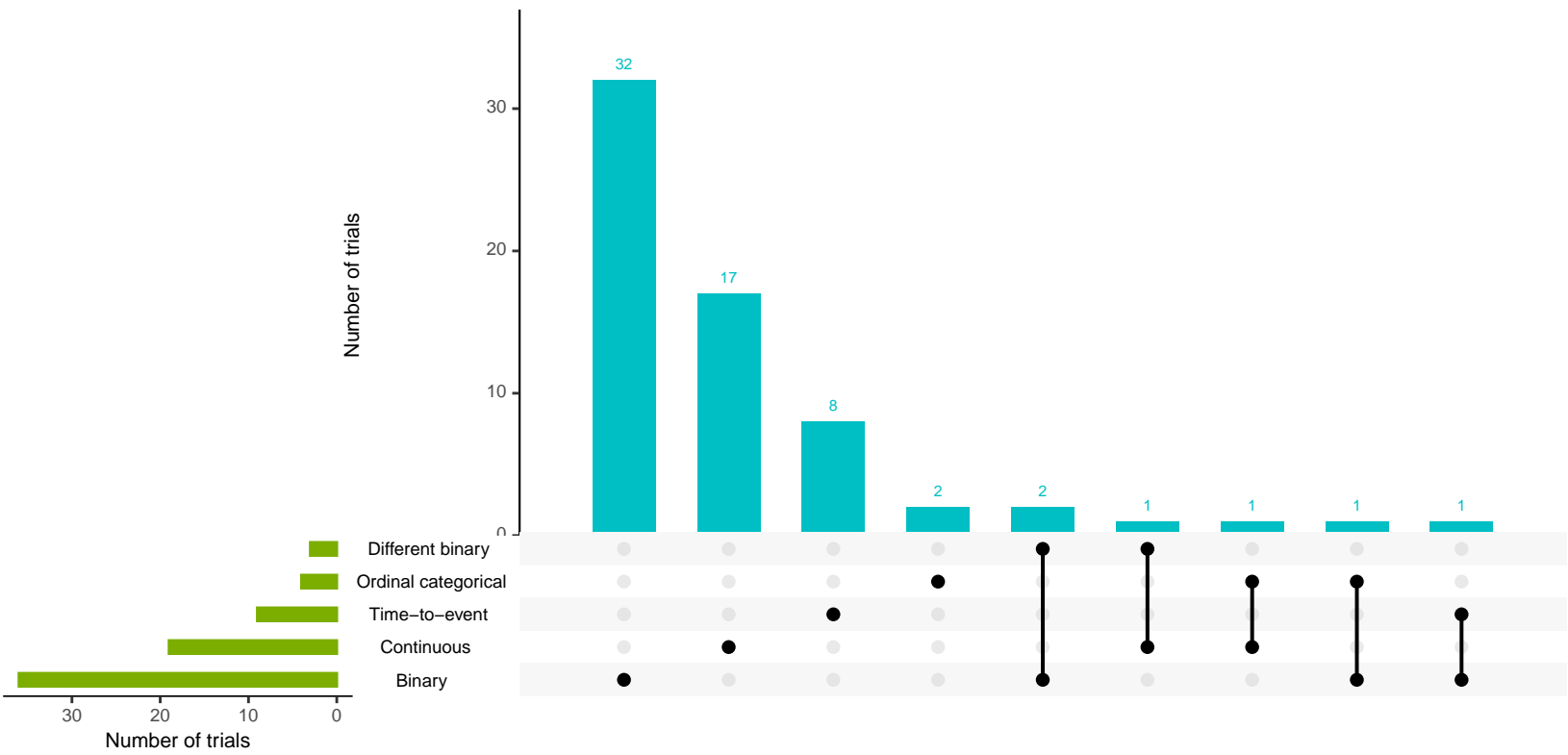

Supplement: sj-pdf-1-smm-10.1177_09622802251348183 - Supplemental material for Response adaptive randomisation in clinical trials: Current practice, gaps and future directions [file sj-pdf-1-smm-10.1177_09622802251348183.pdf]
