# Supplementary material for: Response adaptive randomisation in clinical trials: Current practice, gaps and future directions
Source: Stat Methods Med Res. 2025 Jun 18;34(9):1851–74. doi: 10.1177/09622802251348183 (PMC12460923; doi:10.1177/09622802251348183)
Supplement: sj-pdf-2-smm-10.1177_09622802251348183 - Supplemental material for Response adaptive randomisation in clinical trials: Current practice, gaps and future directions [file sj-pdf-2-smm-10.1177_09622802251348183.pdf]

Supplementary materials

Data items

Table 9: Data items

Master variable	Variable
General	Identification (e.g., webpage)
	Trial ID (e.g., NCT number)
	Source (from search 1, 2, or 3)
	Protocol available (or similar)
	SAP available (or statistical methods described well elsewhere)
Overall & geographical	Recruitment status (completed, in progress, not yet started, terminated, unclear)
	Funder
	Registration/start year
	Disease area
	Location of lead investigator
Design	Trial design
	Trial phase
	Nature of blinding (if blinded, specify who)
	Nature of primary hypothesis
	Trial adaptations considered alongside RAR
	Age group of participants
	Number of treatment arms (including comparator(s))
	Nature of treatment(s)
	Nature of comparator(s)
	Type of primary outcomes
	Time (days) to observe primary outcomes
	Number and/or timing of interim analyses
	Outcome used for the interim analyses different from the primary outcome? (if yes, specify type and time)
	Aspects about confidentiality of interim data clearly described?
	Who made interim decisions/recommendations clearly described?
	Information provided relating to missing data at interim analyses? (if yes, specify approach)
Operating characteristics	Planned maximum, minimum, and expected sample size
	Power & type I error rate
	Methods used to calculate sample size and operating characteristics
	If Bayesian methods were used, were priors described and justified
	Operating characteristics evaluated under different scenarios? (if yes, specify)
RAR algorithm	Main method used & lay description (reference, if applicable)
	Population-level applied at
	Frequency for updating RAR
	Employed a burn-in period? (if yes, give timing, method, allocation ratio)
	Statistical information fed into the algorithm?
	Allocation to arms restricted? (if yes, give details)

Master variable	Variable
	Incorporate safety data?<br>Adjusted for covariates?
Decision-making criteria	Nature of rule(s) (linked to other trial adaptations considered)<br>Timing of the rule(s) different to that of updating the RAR?<br>Criteria for claiming evidence at the end of the trial clearly described?
Results	Nature of results presented<br>Was RAR actually used?<br>Baseline data presented at each interim analysis?<br>Interim analyses conducted at times specified?<br>Actual sample size<br>Changes in allocation ratio reported over time?<br>Other adaptations reported over time?<br>Time trends accounted for? (if yes, give details)<br>Final proportion of participants recruited to treatment arm(s) deemed to have the highest & lowest efficacy relative to control<br>Was what was carried out consistent with descriptions at design stage?<br>If decision-making criteria were specified for other trial adaptations, was it done in line with what was described?<br>If decision-making criteria were specified for claiming evidence at the end of the trial, was it done in line with what was described?<br>Reasons for early stopping (if applicable)
Discussions & resources	Specific concerns discussed relating to RAR<br>Specific areas of future research discussed relating to RAR<br>Links to code, package, or software used for RAR

Trials outside search and trials not yet funded

Table 10 details the trials that were identified outside of the search window (post the 20th of October 2023) and trials that have not yet been funded (by the 20th of October 2023).

Table 10: Trials identified outside of the search window and trials that have not yet been funded

Trial	Link	Type
Empirical Meropenem Versus Piperacillin/Tazobactam for Adult Patients With Sepsis (EMPRESS)	https://clinicaltrials.gov/study/NCT06184659	Outside window
An Adaptive Clinical Trial Design to Identify the Target Dose of Tenecteplase for Treatment of Acute Pulmonary Embolism	https://doi.org/10.1177/17407745221105897	Not yet funded
Intensive Care Platform Trial (INCEPT)	https://incept.dk/	Outside window

Supplemental figures and tables

[INSERT FIGURE 13]

Figure 13: Visualisation of trial primary outcomes and combinations

Table 11: Details of trials where the outcome used for interim analyses was different to the outcome used for primary analyses

Trial	Type of primary outcome	Type of interim outcome	Time to observe primary	Time to observe interim	Further details
ASTIN	Continuous	Continuous (same)	90 days	Early response data	Longitudinal model, based on early response data, used to predict "expected" day 90 score, allowing updates to estimated dose-response relationship before actual scores were collected. Once the actual scores became available, they were used to further update the model.
endTb	Binary	Binary (different)	73 weeks	8 and 39 weeks	Adaptation of probabilities based on interim analyses of two secondary endpoints at 8 and 39 weeks.
INSIGhT	Time-to-event	Time-to-event (different)	2 years	Earlier time points	Primary was overall survival and interim was progress-free survival. Involved less risk of delayed reporting and captured signals earlier.
StratosPHere 2	Continuous	Binary	8 weeks	8 weeks	Interim was binary assuming value 1 if arm showed a change of at least 30% in primary, and 0 otherwise.

List of included trials

Table 12: Trial identifier and web link for trials included in this review

Trial Identifier	Link	Platform?
ACTRN12618000789268	https://anzctr.org.au/ACTRN12618000789268.aspx	No
ACTRN12619000557134	https://www.anzctr.org.au/ACTRN12619000557134.aspx	No
ACTRN12620000408987	https://anzctr.org.au/ACTRN12620000408987.aspx	No
ACTRN12621001223820	https://anzctr.org.au/ACTRN12621001223820.aspx	No
ACTRN12623000024640	https://anzctr.org.au/ACTRN12623000024640.aspx	No
ACTRN12623001004651	https://anzctr.org.au/ACTRN12623001004651.aspx	No
EUCTR 2013-001203-36	https://www.clinicaltrialsregister.eu/ctr-search/trial/2013-001203-36/PT	No
ISRCTN10304915	https://doi.org/10.1186/ISRCTN10304915	No
ISRCTN86534580	https://doi.org/10.1186/ISRCTN86534580	Yes
ISRCTN96528723	https://doi.org/10.1186/ISRCTN96528723	No
NCT00142571	https://clinicaltrials.gov/study/NCT00142571	No
NCT00409968	https://clinicaltrials.gov/study/NCT00409968	No
NCT00500578	https://clinicaltrials.gov/study/NCT00500578	No
NCT00555204	https://clinicaltrials.gov/study/NCT00555204	No
NCT00915005	https://clinicaltrials.gov/study/NCT00915005	No
NCT01000051	https://clinicaltrials.gov/study/NCT01000051	No
NCT01042379	https://clinicaltrials.gov/study/NCT01042379	Yes
NCT01248247	https://clinicaltrials.gov/study/NCT01248247	No

Trial Identifier	Link	Platform?
NCT01289457	https://clinicaltrials.gov/study/NCT01289457	No
NCT01369069	https://clinicaltrials.gov/study/NCT01369069	No
NCT01391338	https://clinicaltrials.gov/study/NCT01391338	No
NCT01613586	https://clinicaltrials.gov/study/NCT01613586	No
NCT01665092	https://clinicaltrials.gov/study/NCT01665092	No
NCT01767311	https://clinicaltrials.gov/study/NCT01767311	No
NCT01769820	https://clinicaltrials.gov/study/NCT01769820	No
NCT01786343	https://clinicaltrials.gov/study/NCT01786343	No
NCT01873404	https://clinicaltrials.gov/study/NCT01873404	No
NCT01960075	https://clinicaltrials.gov/study/NCT01960075	No
NCT01995838	https://clinicaltrials.gov/study/NCT01995838	No
NCT02045797	https://clinicaltrials.gov/study/NCT02045797	No
NCT02110069	https://clinicaltrials.gov/study/NCT02110069	No
NCT02166333	https://clinicaltrials.gov/study/NCT02166333	No
NCT02260388	https://clinicaltrials.gov/study/NCT02260388	No
NCT02380625	https://clinicaltrials.gov/study/NCT02380625	Yes
NCT02407028	https://clinicaltrials.gov/study/NCT02407028	No
NCT02508649	https://clinicaltrials.gov/study/NCT02508649	No
NCT02556606	https://clinicaltrials.gov/study/NCT02556606	No
NCT02626299	https://clinicaltrials.gov/study/NCT02626299	No
NCT02735707	https://clinicaltrials.gov/study/NCT02735707	Yes
NCT02754765	https://clinicaltrials.gov/study/NCT02754765	No
NCT02977780	https://clinicaltrials.gov/study/NCT02977780	Yes
NCT03021928	https://clinicaltrials.gov/study/NCT03021928	No
NCT03201419	https://clinicaltrials.gov/study/NCT03201419	No
NCT03227224	https://clinicaltrials.gov/study/NCT03227224	No
NCT03227328	https://clinicaltrials.gov/study/NCT03227328	No
NCT03251482	https://clinicaltrials.gov/study/NCT03251482	No
NCT03596762	https://clinicaltrials.gov/study/NCT03596762	No
NCT03896763	https://clinicaltrials.gov/study/NCT03896763	No
NCT03903835	https://clinicaltrials.gov/study/NCT03903835	Yes
NCT03970447	https://clinicaltrials.gov/study/NCT03970447	Yes
NCT04195256	https://clinicaltrials.gov/study/NCT04195256	No
NCT04217551	https://clinicaltrials.gov/study/NCT04217551	No
NCT04229758	https://clinicaltrials.gov/study/NCT04229758	No
NCT04344587	https://clinicaltrials.gov/study/NCT04344587	No
NCT04372589	https://clinicaltrials.gov/study/NCT04372589	No
NCT04790786	https://clinicaltrials.gov/study/NCT04790786	Yes
NCT05137119	https://clinicaltrials.gov/study/NCT05137119	Yes
NCT05758246	https://clinicaltrials.gov/study/NCT05758246	No

**Trial Identifier**	**Link**	**Platform?**
NCT05913622	https://clinicaltrials.gov/study/NCT05913622	Yes
Unknown	10.1161/01.STR.0000092527.33910.89	No
Unknown	10.1542/peds.76.4.479	No
Unknown	10.1200/JCO.2003.11.016	No
Unknown	10.1002/cncr.29085	No
Unknown	10.1186/s13063-022-06664-4	Yes
Unknown	10.1097/AAP.0b013e31820d4266	No
